# Aqueous Phase Hydrogenation of 4-(2-Furyl)-3-buten-2-one over Different Re Phases

**DOI:** 10.3390/molecules29163853

**Published:** 2024-08-14

**Authors:** Claudio Ignacio C. Díaz, Claudio Araya-López, A. B. Dongil, Nestor Escalona

**Affiliations:** 1 Departamento de Ingeniería Química y Bioprocesos, Escuela de Ingeniería, Pontificia Universidad Católica de Chile, Avenida Vicuña Mackenna 4860, Macul, Santiago 7820436, Chile; cicontreras6@uc.cl (C.I.C.D.); claudio.arayal@uc.cl (C.A.-L.); 2 Millenium Nuclei on Catalytic Processes towards Sustainable Chemistry (CSC), Santiago 7820436, Chile; 3 Instituto de Catálisis y Petroleoquímica, CSIC, Cantoblanco, 28049 Madrid, Spain; 4 Departamento de Química Física, Facultad de Química y de Farmacia, Pontificia Universidad Católica de Chile, Santiago 7820436, Chile

**Keywords:** hydrogenation, rhenium, 4-(2-furyl)-3-buten-2-one, Piancatelli rearrangement

## Abstract

4-(2-furyl)-3-buten-2-one (FAc) is obtained by aldol condensation of furfural and acetone and has been used in hydrodeoxygenation reactions to obtain fuel products using noble metal catalysts. The hydrogenation of FAc in the aqueous phase using metallic- and Re oxide-supported catalysts on graphite was studied, within a temperature range of 200–240 °C, in a batch reactor over a 6 h reaction period. The catalysts were characterized using N_2_ adsorption–desorption, TPR-H_2_, TPD-NH_3_, XRD, and XPS analyses. Catalytic reactions revealed that metallic rhenium and rhenium oxide-supported catalysts are active for the hydrogenation and Piancatelli rearrangement of FAc. Notably, metallic rhenium exhibited a fourfold higher initial rate than rhenium oxide, which was attributed to the higher dispersion of Re in the Re/G catalyst over graphite. Re/G and ReOx/G catalysts tended to rearrange and hydrogenate FAc to 2-(2-oxopropyl)cyclopenta-1-one in water.

## 1. Introduction

Energy supply worldwide has traditionally relied heavily on fossil fuels, which are depleting and limited by their geographical distribution and ease of extraction [1]. As we face the challenges of dwindling fossil fuel resources and the need for more sustainable energy solutions, attention has turned to alternative sources and innovative processes. One such promising avenue is the utilization of hemicellulose, a renewable and versatile biopolymer found in plant cell walls. Unlike lignin, which tends to yield phenolic compounds upon decomposition, hemicellulose undergoes conversion during processing, resulting in higher production of relatively small, oxygenated compounds. This unique characteristic makes hemicellulose a valuable feedstock with diverse applications, including biofuels, chemicals, and other value-added products [2,3,4].

The production of biofuels faces a significant challenge due to the high oxygen content of biomass-derived molecules, impacting their quality and compatibility with conventional fuels. Biofuels benefit from addressing this issue by combining various compounds, including linear hydrocarbons, cyclic hydrocarbons, olefins, and aromatics [5,6]. Driven by the ongoing pursuit of sustainable energy solutions, hydrodeoxygenation (HDO) has proven instrumental in enhancing biofuels by reducing the oxygen content [7,8]. This, in turn, increases the heating value, improves compatibility with fossil fuels, and mitigates acidity. However, the conventional reliance on high-pressure hydrogen in HDO introduces challenges, particularly concerning the expensive transportation and storage of gaseous hydrogen [9].

As the most efficient method of raising the very low combustion enthalpy of condensation adducts such as 4-(2-furyl)-3-buten-2-one, deoxygenation can decrease unsaturation and oxygen content [10,11]. Due to its excellent performance in the hydrodeoxygenation steps and ring opening to obtain n-octane, Pt catalysts have been extensively studied along this process. Pt is an expensive noble metal, and it is known that HDO of FAc is exceedingly difficult due to its wide range of distinct products aside from the deoxygenated products [12]. To achieve more specialized value-added goods at reduced preparation costs, new materials are therefore required.

Graphite, as a highly anisotropic mineral, demonstrates thermal and electrical conductivities roughly 1000 times greater in the axial direction compared to the direction perpendicular to its plane. It is the most stable allotrope of carbon, characterized by bonds exhibiting sp2 hybridization. Structurally, it consists of graphene sheets stacked and bonded through π-π stackings or van der Waals molecular forces, resulting in a sandwich-like structure [13]. High surface area graphite (HSAG) is generally used in heterogeneous catalysis as support due to its remarkable surface area [14,15,16,17].

Previous studies have extensively explored the catalytic properties of rhenium [18] in various phases, including metallic [19], oxide [20], carbide [21], and sulfide [22], demonstrating its prominence as a catalyst in the HDO reactions of compounds such as guaiacol and furfural. Rhenium-based catalysts in aqueous-phase hydrogenations of 4-(2-furyl)-3-buten-2-one have not yet been reported.

As extensively discussed in existing literature, the hydrogenation and rearrangement of furfurals into cyclic compounds involve a series of stages. These encompass the hydrogenation of C = O bonds, the rearrangement of the ring structure, hydrogenation, and subsequent dehydration. These transformations occur on reduced metals or solid acids within a water-based solvent, operating under an H_2_ pressure from 2.0 to 8.0 MPa and at temperatures between 140 and 180 °C [23].

On the other hand, 4-(2-furyl)-3-buten-2-one has been extensively used as a model molecule to obtain jet-fuel products due to its derivation from the aldol condensation of furfural and acetone. For this reason, the HDO-ring opening of this molecule is of main concern to improve the energy content of biofuels [24,25,26].

The present research was studying the use of Re in metallic and oxide phases supported on graphite in the hydrogenation and rearrangement of 4-(2-furyl)-3-buten-2-one in aqueous phase.

## 2. Results and Discussion

### 2.1. Characterization of Catalysts

**Figure 1:**
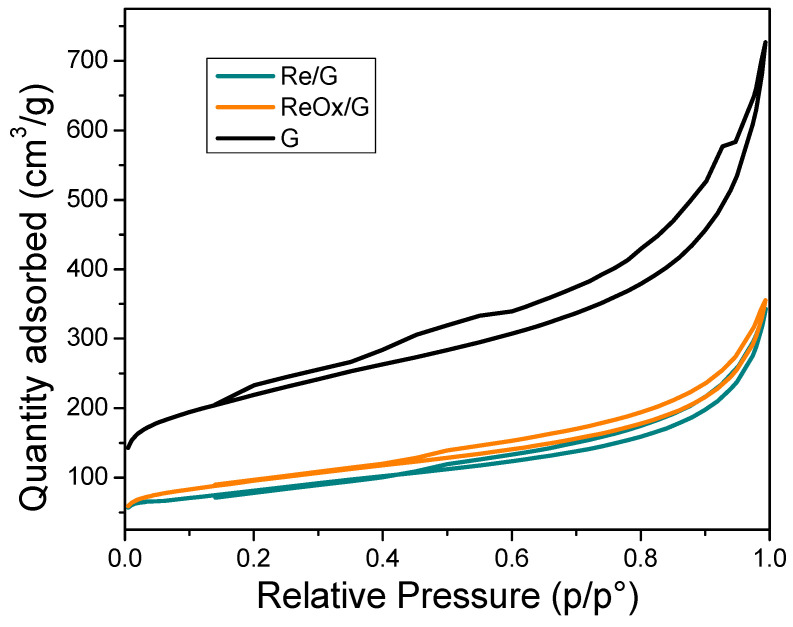
Isotherm of N_2_ adsorption/desorption of rhenium-based catalyst supported on graphite.

Figure 1 shows the isotherm of N_2_ adsorption/desorption of rhenium-based catalyst supported on graphite. All catalysts in Figure 1 displayed a type IV isotherm with an H4 hysteresis loop according to IUPAC criteria [27], representative of mesoporous and slit-shape pores, respectively, and representative of graphite materials [28]. Table 1 summarizes the textural properties obtained from Figure 1, which shows a diminution of surface area, pore volume (total, micro, and mesoporous), and the increase in the pore diameter of all catalysts. This behavior suggests a partial pore blocking with 10% rhenium. Figure S1 shows that the pore size distribution confirms the filling of the pores due to the decrease in the dV/dlogw.

**Figure 2:**
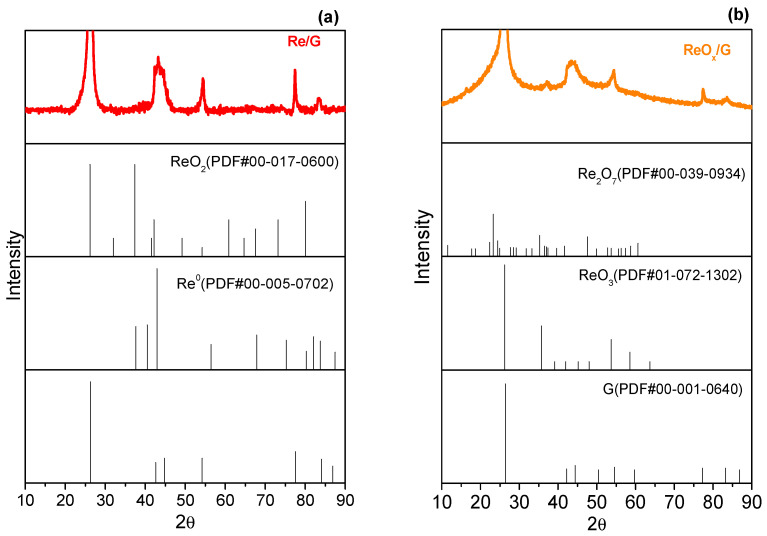
XDR patterns of (**a**) Re/G and (**b**) ReOx/G catalysts diffractograms.

The XRD patterns for each catalyst are depicted in Figure 2. All catalysts exhibit distinctive diffraction peaks of carbon at approximately 2θ = 26°, 43°, and 55°, corresponding to the (002), (100)/(101), and (004) planes, respectively. Notably, for ReO_x_/G and Re/G catalysts, diffraction peaks related to rhenium species are barely visible, suggesting the formation of particle sizes near the detection limit of the equipment (≤4 nm) [29]. In the case of Re/G (Figure 2a), the most prominent diffraction peak at 42° is observed, which overlaps with the main diffraction of graphite, and is attributed to the HCP-Re phase (ICCS, PDF no. 00–005–0702). Figure 2b shows a low-intensity peak at 35° and it was identified as ReO_3_.

The TPD-MS-He profile of graphite is displayed in Figure S2. The CO_2_ profile presents a main broad peak at 318 °C with two shoulders at 245 °C and 450 °C. This result suggests the presence of different types of chemical species on the surface as carboxylic acid or anhydride groups. The evolution of H_2_O (*m*/*z* = 18) at 218 °C followed by the simultaneous evolution of CO_2_ and CO at 242–245 °C indicate the formation of anhydride groups during the experiment, which then decompose as CO_2_ and CO at higher temperatures [30]. The CO profile (*m*/*z* = 28) shows a wide peak centered at 630 °C with a shoulder at 242 °C that could be assigned to phenolic and anhydride groups [31]. 

**Figure 3:**
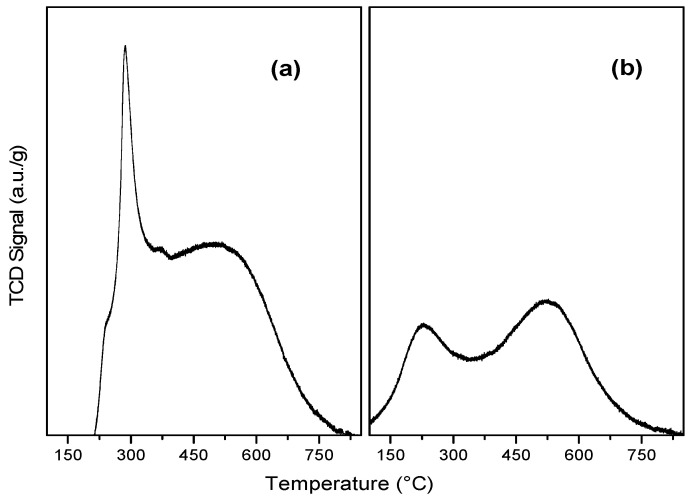
Temperature-programmed reduction of calcined (**a**) ReOx/G and reduced (**b**) Re/G catalysts supported on graphite with TCD-signal.

In Figure 3a, the ReOx/G catalyst exhibits an intense reduction peak at 284 °C and a broad peak centered at 500 °C. The first reduction peak was attributed to the reduction of rhenium oxide species [32,33]. The second reduction peak was assigned to the reduction of the support, in agreement with the CH_4_, CO, and CO_2_-MS signals (Figure S3).

In Figure 3b, the Re/G catalyst shows a low-intensity reduction peak centered at 200 °C and another at 500 °C. The first reduction peak was assigned to the reduction of rhenium oxide, suggesting that there is no complete reduction to metallic rhenium, in accordance with what was observed by XPS. The second reduction peak was ascribed to the reduction of the support, similar to that observed in ReOx/G.

**Figure 4:**
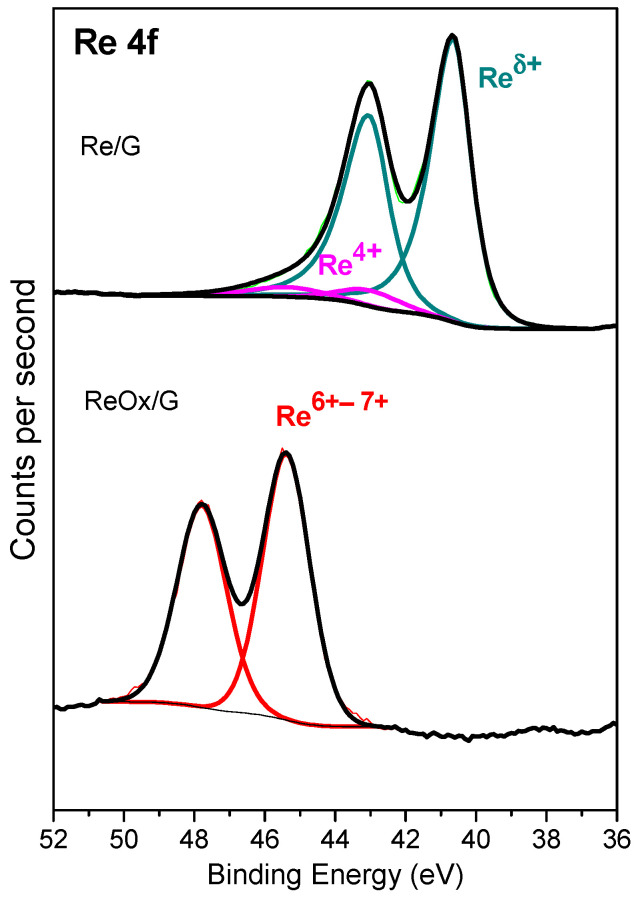
Re 4f core levels spectra of the rhenium-based catalysts.

Figure 4 shows XPS analysis in the Re 4f region of the catalysts. The Re/G catalyst displayed two overlapping doublets, each one containing the Re 4f_7/2_ and 4f_5/2_ peaks, while the ReOx/G catalyst presented only one doublet. Table 2 summarizes the binding energies of the most intense Re 4f_7/2_ component of each doublet, as well as the atomic surface ratio of Re/C, O/Re, and O/C. The peak at 40.6 eV in the Re 4f_7/2_ region is ascribed to Re^δ+^, which could be associated with both rhenium carbide and metallic rhenium [34,35]. The presence of the metallic rhenium observed by XPS agrees with the XRD pattern. The Re/G catalyst displayed a peak at a BE of 43.0 eV that corresponds to Re^4+^ on ReO_2_ [36], concordant with TPR of the reduced sample. ReO_2_ is not observed in X-ray diffraction, suggesting a high dispersion over graphite. On the other hand, the ReOx/G catalyst displayed a peak at a BE of 45.4 eV which could be attributed to Re^6+^ in agreement with XRD. However, Leiva et al. [22] suggested that the BE at 46 eV could be attributed to Re^7+^ with oxygen vacancies. Nonetheless, the presence of ReO_4_^−^ was not observed by XRD, confirming a high dispersion of this species on the surface with a low particle size. 

The surface atomic ratios of rhenium samples in Table 2 show that the Re/G sample has a Re/C ratio of almost five times that of ReOx/G, suggesting that the metallic rhenium catalyst has a higher dispersion of Re on the support; similar behavior was observed previously by Leiva et al. [22]. 

On the other hand, the O/Re atomic ratio of ReOx/G is seven times higher than Re/G catalyst, consistent with oxide species. 

**Figure 5:**
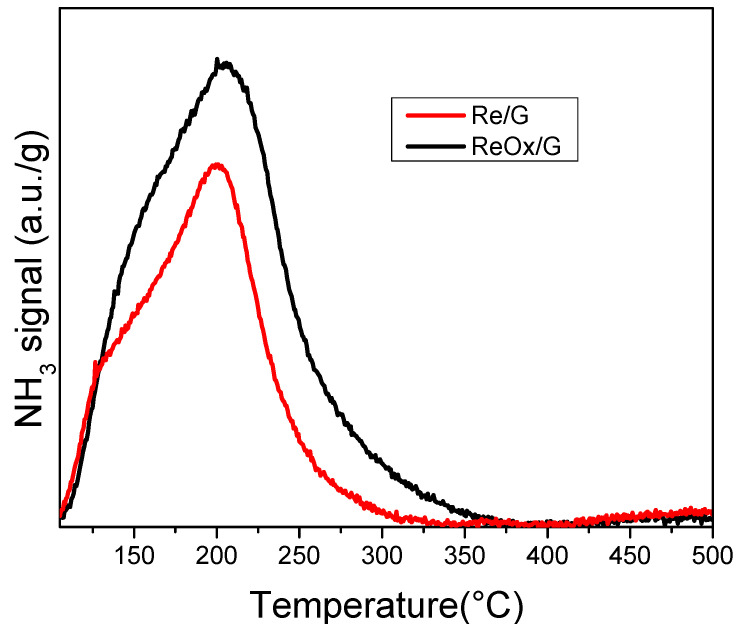
Temperature-programmed desorption-NH_3_ of rhenium-based catalysts.

Figure 5 shows the TPD-NH_3_ profiles of the catalysts. For both catalysts a well-defined NH_3_ desorption peak at about 200 °C is observed and assigned to weak acid sites, characteristics of rhenium species [19,37]. 

The quantity of total acid sites is summarized in Table 3 for Re/G and ReO_x_/G catalysts. ReOx/G possesses a higher amount of acid sites than Re/G, this could be attributed to Lewis acid sites created in the process of formation of vacancies suggested by XPS. 

### 2.2. Catalytic Activity

**Figure 6:**
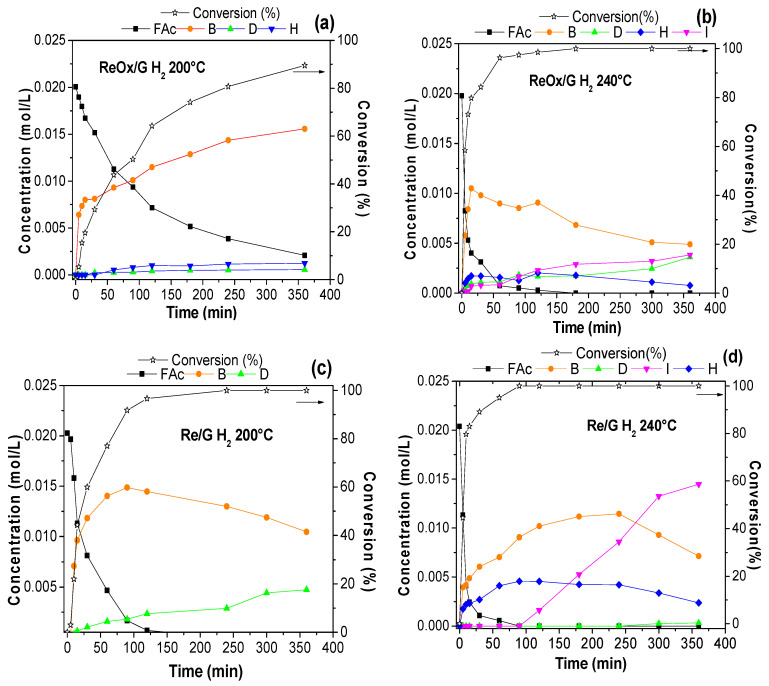
Conversion and kinetics of 4-(2-furyl)-3-buten-2-one with (**a**) ReOx/G at 200 °C (**b**) ReOx/G at 240 °C, (**c**) Re/G at 200 °C, and (**d**) Re/G at 240 °C.

The aqueous-phase hydrogenation of Fac over rhenium-based catalysts is shown in Figure 6. For a comprehensive understanding, the reaction products were labeled as: 4-(2-furanyl)butan-2-one (B) as the product of the hydrogenation of the C = C bond; 4-(2-furanyl)butan-2-ol (D) as the product of hydrogenation of C = C and C = O; and 2-(2-oxopropyl)cyclopent-2-en-1-one (H) and 2-(2-oxopropyl)cyclopentane-1-one (I) for both ring-rearrangement products. Regarding the ReOx/G catalyst, Figure 6a shows that ‘B’ is the major product, while ‘H’ and ‘D’ are in minor quantities at 200 °C. However, ‘B’ in Figure 6b is observed as a major product followed by ‘H’, ‘D’, and ‘I’; then, ‘B’, and ‘H’ slightly decrease as ‘I’ and ‘D’ increase with time. 

Figure 6c, where the reaction is carried out at 200 °C over Re/G, it is observed that Fac is hydrogenated to turn into ‘B’, which has a steep production at the beginning of the reaction until reaching a maximum at a reaction time of 90 min, then ‘B’ decreases over time. In the meantime, ‘D’ shows a gradual increase as the reaction progresses, indicating that ‘B’ is an intermediary to produce ‘D’. When the reaction was carried out at 240 °C, the product distribution significantly changed as can be seen in Figure 6d. There is barely any formation of ‘D’ product, and ‘B’ and ‘H’ seem to be intermediaries for ‘I’, which clearly shows a considerable increase after 90 min of reaction. It is noticed that ‘I’ is the final product in this reaction. 

**Figure 7:**
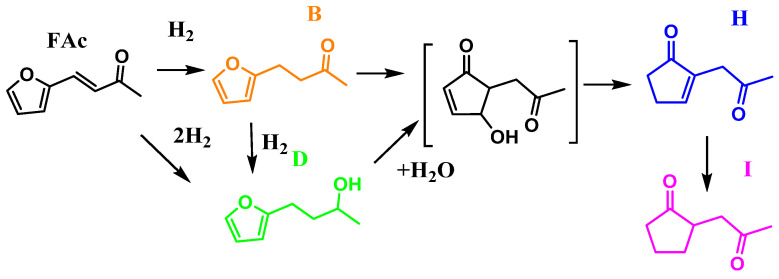
Proposed reaction network. 4-(2-furanyl)butan-2-one (Orange); 4-(2-furanyl)butan-2-ol (Green); 2-(2-oxopropyl)cyclopent-2-en-1-one (Blue) and 2-(2-oxopropyl)cyclopentane-1-one (Magenta).

Based on the evolution of the products as a function of time (Figure 6), Figure 7 shows the proposed reaction network. The first reaction step is the hydrogenation of the C = C bond of Fac to produce ‘B’, followed by a second hydrogenation over the C = O bond to give ‘D.’ Nevertheless, it is expected that ‘D’ is not only obtained by the hydrogenation of ‘B’ but also by the straight hydrogenation of Fac. 

To produce ‘H’ and ‘I’, the structure of the molecule changed from a furan ring into cyclopentanone-derived molecules. In Figure 7, either ‘B’ or ‘D’ passes through hydration and then a Piancatelli rearrangement, which is usually an acid-catalyzed reaction carried out in aqueous-phase media. The proposed intermediary may explain the conversion from furan rings into cyclopentanone-derived molecules (H, and I). Furthermore, the final product in the reaction network is ‘I’, as identified by the mass spectrometer depicted in Figure S4. 

The furan ring rearrangement was observed before for furfural [38] and 5-hydroxymethylfurfural [39] molecules in aqueous phase. In addition, surface Brønsted acidity could be formed during the reaction by the interaction of water molecules and Lewis acid sites, thus improving the catalytic hydrogenation of the insaturations in the cyclopentanone ring. 

Table 4 summarizes the initial rates obtained from Figure S1. The Re/G catalyst displayed higher activity than ReOx/G in both systems at 200 °C and 240 °C. The initial rate of hydrogenation of 4-(2-furyl)-3-buten-2-one over ReOx/G was 2.3 times slower than Re/G at 200 °C, while at 240 °C the initial rate was 1.2 times slower. This behavior could be attributed to an increase in the oxygen vacancies of the ReOx/G with temperature favoring the creation of new active sites. In fact, Ghampson et al. [40] explained that the catalytic acid sites on rhenium oxides are coordinatively unsaturated metal sites.

The higher activity of Re/G over ReOx/G could be attributed to the higher surface concentration of Re over the graphite surface, as suggested by the XPS results. Table S1 shows a comparison of the initial rates in this work compared to the literature.

**Figure 8:**
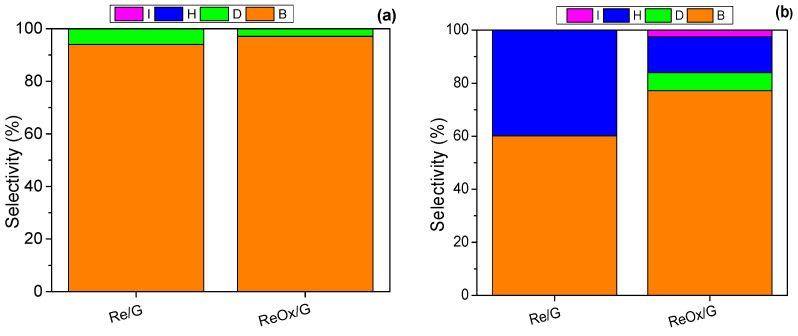
Product selectivity at (**a**) 200 °C and (**b**) 240 °C at 40% conversion.

Figure 8 shows the selectivity of the catalysts at 40% conversion. In Figure 8a, it is observed that both catalysts show the same reaction products (‘B’ and ‘D’) at 200 °C. This suggests that the reaction mechanism may be similar in both catalysts at 200 °C. 

On the other hand, Figure 8b shows that in the Re/G and ReOx/G catalysts at 240 °C the product distribution changes significantly. The Re/G catalyst forms ‘B’ and ‘H’ products, while ReOx/G produces ‘B’ followed by ‘H’, ‘D’, and ‘I’; these results suggest that the reaction mechanism between both catalysts is different [13]. Furthermore, the formation of furyl-rearrangement products (‘H’ and ‘I’) required a hydrogenation step. This behavior suggests the ReOx/G catalyst favors a higher hydrogenation than the Re/G catalyst. On the other hand, these vacancies promote the heterolysis of H_2_ on the catalyst surface, followed by the subsequent formation of Brønsted acid sites upon contact with water [41].

**Figure 9:**
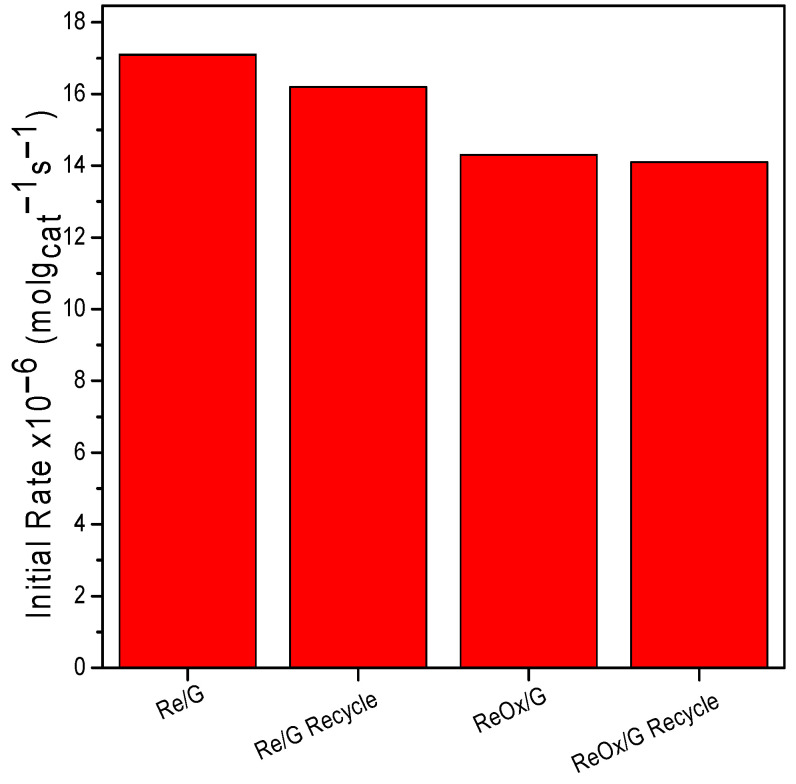
Initial rate of rhenium-based catalysts and recycles at 2 h of reaction.

Figure 9 shows the recycling of both catalysts used in the hydrogenation of furfural-acetone. The catalyst was recovered and either reduced or calcined depending on the initial treatment. It is observed that the initial rate corresponding to Re/G decreases, which may be due to the sintering of Re on the support surface. This phenomenon was previously reported using Re/SiO2 by Leiva et al. [22]. However, we cannot rule out the presence of leaching, even though this phenomenon has been reported at reaction times of 24 h for the liquid phase and 30 h for the gas phase, as reported by Harth et al. [42]. Meanwhile, ReOx/G maintains its activity, suggesting that under these reaction conditions the catalyst does not deactivate.

## 3. Materials and Methods

### 3.1. Catalysts Preparation

The catalysts were prepared by incipient wetness impregnation with an aqueous solution of NH_4_ReO_4_ (Molymet S.A.) on a high surface area of graphite (Timcal Graphite, 400 m^2^/g), denoted as G. All catalysts were prepared with 10% of metal loading. The impregnated catalyst was aged for 3 h at room temperature, dried at 120 °C, and treated at different conditions. One part of the catalyst was treated with H_2_ (50 mL/min) at 350 °C for 1 h with a ramp of 5 °C/min and labeled Re/G, and another portion was treated with N_2_ (50 mL/min) at 350 °C for 1 h with a ramp of 5 °C/min and labeled as ReOx/G.

### 3.2. Characterization of the Catalysts

Utilizing the Micromeritics 3Flex instrument, textural characteristics were evaluated through N_2_ sorption isotherms at 77 K. Before each analysis, 10 mg samples underwent a 4 h degassing process at 300 °C under vacuum using the Micromeritics SmartVacPrep instrument. Surface area calculations are based on the Brunauer–Emmett–Teller (BET) theory using Rouquerol criteria [43]. The determination of the total pore volume was achieved as the single-point pore volume at *p*/p^0^ = 0.99. The micropore volume was calculated by a *t*-plot analysis using the equation of Halsey [44] and pore size distribution was determined with the BJH method using the desorption branch. The analysis of the in situ treated samples by reduction (Re/G) and calcination (ReOx/G) reducibility involved H_2_-temperature-programmed reduction (H_2_-TPR) using a Micromeritics 3Flex instrument coupled with a mass spectrometer (Cirrus 2, MKS Spectra Product) and a thermal conductivity detector (TCD). Samples weighing between 15 and 20 mg were loaded into a quartz reactor tube and heated from room temperature to 1000 °C at a rate of 10 °C min^−1^ in a 5% H_2_-Ar flow (100 mL min^−1^). The resulting gas was directed through a cold trap immersed in a mixture of isopropanol and liquid nitrogen before entering the mass spectrometer and TCD detector. The mass signals correspond to CO (*m*/*z* = 28), CO_2_ (*m*/*z* = 44), and CH_4_ (*m*/*z* = 16). The presented reduction profiles were derived from the calibrated TCD signals for CO, CO_2_, and CH_4_. For the temperature-programmed desorption of NH_3_ (TPD-NH_3_), the sample underwent initial pretreatment for 30 min at 350 °C (10 °C/min) under a flow of He (50 mL/min). Subsequently, the adsorption of NH_3_ was conducted at 100 °C for 15 min (30 mL/min), followed by desorption under He (100 mL/min) at a rate of 10 °C/min until reaching 500 °C. During both H_2_-TPR and TPD-NH_3_ treatments, products formed were identified using a mass spectrometer, specifically by monitoring specific fragments the same as with TPR, including ammonia (*m*/*z* = 17). Using a Polycrystal X’Pert Pro PANalytical diffractometer with Ni-filtered CuKα radiation (λ = 1.54 Å), X-ray diffraction (XRD) patterns of the passivated catalysts were acquired. The measurements were conducted within the 2θ range from 4° to 90°, with a scanning step of 0.04° s^−1^. The XRD instrument operated at 45 kV and 40 mA during data acquisition. XPS measurements were carried out using a PHOIBOS 1509MCD energy analyzer from SPECS GmbH. Re/G was reduced, and ReOx/G was calcined ex situ as previously explained. The instrument employed non-monochromatic Al radiation at 200 W with an energy level of 1486.61 eV. Before the experiments, the samples were compacted into pellets and placed inside a pre-evacuated vacuum chamber. In preparation for the experiments, the samples underwent in situ outgassing for 24 h, achieving a dynamic vacuum level lower than 10^−10^ mbar. The binding energy (BE) was determined by referencing it to the C1s peak at 284.6 eV, with the energy measurements displaying an equipment error of less than 0.01 eV.

### 3.3. Catalytic Tests

The hydrodeoxygenation reactions of 4-(2-furyl)-3-buten-2-one (FAc) were carried out in a 100 mL stainless steel batch reactor manufactured by Parr. In one reaction, the solid reactant was dissolved in 50 mL of solvent. A specific amount of recently reduced or calcined catalyst was introduced directly in reactor, resulting in a reactant-to-catalyst ratio of 24.8. The reactor was purged with nitrogen (N_2_) and agitated at 300 revolutions per minute (RPM) until the desired temperature was reached. 

Once the desired temperature was attained, 30 bars of hydrogen (H_2_) were introduced, and the agitation was increased to 700 RPM (this was taken as the t = 0). Subsequent sample collection was performed through a stainless steel tube at different time intervals until 6 h of reaction passed. Products were identified and quantified by gas chromatography with mass spectrometry with an Agilent 8890. Conversion and product selectivity were defined according to the following formulas:

$$Conversion\left(\%\right)=\frac{n_{FAc}^{0}-n_{FAc}}{n_{FAc}^{0}}\cdot 100$$

$$Selectivity\left(\%\right)=\frac{n_{Product\;i}}{\sum n_{Products}}\cdot 100$$

where $n_{FAc}^{0}$ and $n_{FAc}$ correspond, to the molar quantity measured at t = 0 and t, respectively, and $n_{Product\;}$is the molar quantity of a product *i*.

The initial reaction rate (mol g^−1^ s ^−1^) was calculated from the initial slope (*b*) of the conversion vs. time plot (s^−1^) according to the formula:

$$r_{0}=\frac{b\cdot n_{FAc}^{0}}{m}$$

where *m* (g) is the mass of the catalyst. Carbon balance was over 90%.

## 4. Conclusions

Supported Re in metallic and oxide states was active for hydrogenation of 4-(2-furyl)-3-buten-2-one in water as solvent. 2-(2-oxopropyl)cyclopentan-1-one was produced from a Piancatelli rearrangement obtained from FAc over Re/G and ReOx/G catalysts. The Re/G catalyst displayed higher activity in the hydrogenation of FAc at 200 °C and at 240 °C. The superior performance of Re/G over ReOx/G was attributed to the higher surface distribution of Re over the graphite. Re/G obtained major production of 2-(2-oxopropyl)cyclopentan-1-one. The catalysts displayed similar product distribution calculated at 40% conversion at 200 °C, suggesting a similar reaction mechanism. However, at 240 °C a converse behavior was observed. The changes in the reaction mechanism were attributed to an increment in the vacancies of the ReOx/G. The Re/G catalyst is promising for the production of 2-(2-oxopropyl)cyclopentan-1-one which is a cyclic reactant for alternative aviation fuel. The recycling of the catalysts showed that metallic rhenium exhibits slight deactivation, while rhenium oxide remains constant. These results suggest that rhenium oxide is a promising catalyst for this type of reaction.

## Figures and Tables

**Table 1:** Textural properties obtained for bare supports and rhenium-based catalysts.

Samples	S_BET_ (m^2^/g)	V_p_ (cm^3^/g)	V_m_ (cm^3^/g)	V_µ_ (cm^3^/g)	Dp (nm)
G	430	0.87	0.841	0.040	5.9
ReOx/G	331	0.54	0.513	0.027	6.5
Re/G	283	0.51	0.488	0.022	7.3

**Table 2:** XPS binding energies(eV) and surface atomic ratios of Re 4f for the rhenium-based catalysts.

		Phases	Re/G	ReOx/G
BE, eV (%)	Re 4f7/2	Re^δ+^	40.6 (93)	-
		Re^4+^	43.0(7)	-
		Re^6–7+^	-	45.4 (100)
Re/C Ratio	-		0.0346	0.0067
O/Re Ratio			0.92	6.8

**Table 3:** Total acid sites of rhenium-based catalysts.

Samples	Total Acid Sites (10^−5^ mol NH_3_·g^−1^)
Re/G	1.1
ReOx/G	1.9

**Table 4:** Initial rate and operational condition of the experiments with rhenium-based catalysts.

Samples	Reaction Temperature	r_0_ (×10^−6^ mol·gcat^−1^·s^−1^)
Re/G	200	4.41
ReOx/G		1.88
Re/G	240	17.3
ReOx/G		14.05

## Data Availability

Data are contained within the article or Supplementary Material.

## Supplementary Materials

The following supporting information can be downloaded at: https://www.mdpi.com/article/10.3390/molecules29163853/s1, Figure S1: Pore size distribution for Re-based catalysts; Figure S2: TPD-He of high surface graphite support; Figure S3: MS-signals of (a) CH_4_, (b) CO_2_, (c) CO observed during the TPR; Table S1: Initial rate of hydrogenation of FAc; Figure S4: mass spectra of ring rearrangement products [45].
