# Supplementary material for: Aqueous Phase Hydrogenation of 4-(2-Furyl)-3-buten-2-one over Different Re Phases
Source: Molecules. 2024 Aug 14;29(16):3853. doi: 10.3390/molecules29163853 (PMC11357411; doi:10.3390/molecules29163853)
Supplement: Supplementary file 1 [file molecules-29-03853-s001.zip › molecules-3109565-supplementary.pdf]

Article

Aqueous phase hydrogenation of 4-(2-furyl)-3-buten-2-one over different Re phases

Claudio Ignacio C. Díaz [1,2] , Claudio Araya-López [1,2], A. B. Dongil [3,*] and Nestor Escalona [1,2,4,*]

[1] Departamento de Ingeniería Química y Bioprocesos, Escuela de Ingeniería, Pontificia Universidad Católica de Chile, Avenida Vicuña Mackenna 4860, Macul, Santiagocicontreras6@uc.cl (C.I.C.D.); claudio.arayal@uc.cl (C.A.-L.)

[2] Departamento de Química Física, Facultad de Química y de Farmacia, Pontificia Universidad Católica de Chile, 7820436 Santiago, Chile;

[3] Instituto de Catálisis y Petroleoquímica, CSIC, Cantoblanco, 28049 Madrid, Spain

[4] Millenium Nuclei on Catalytic Processes Towards Sustainable Chemistry (CSC), Chile

* Correspondence: a.dongil@csic.es (A.B.D.); neescalona@uc.cl (N.E.)

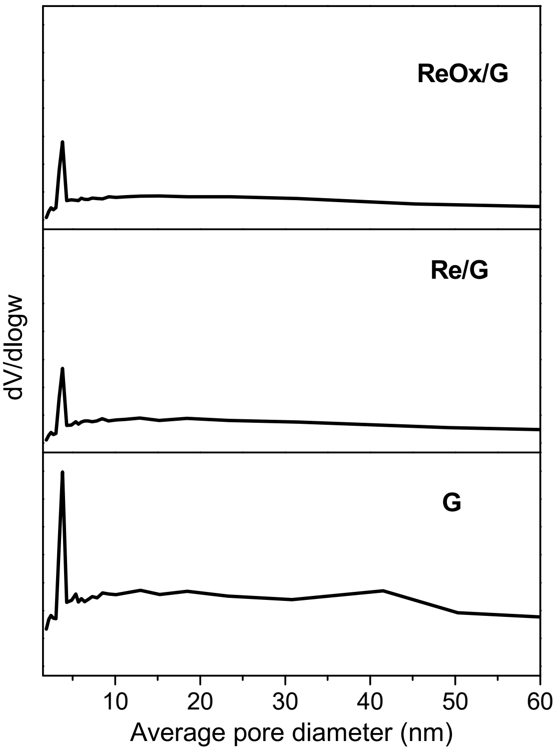

Figure S1. Pore size distribution for Re-based catalysts

Figure S1 shows an intense peak at the beginning, followed by a gradual decrease. This initial peak is attributed to an artifact of the technique, which is formed at the moment of closing the hysteresis loop and cannot be considered as a pore of the material.

According to the literature, oxygen-formed groups (OFG) could affect the metal-support interaction between rhenium and graphite[22]. H_2-TPR measurements were carried out to discuss and observe the interaction between rhenium and graphite, and the effect of reduction on functional groups presented on the graphite surface [28]. The profile displays signals corresponding to desorbing species at specific mass-to-charge ratios (m/z): m/z = 44 (assigned to CO_2), m/z = 28 (assigned to CO), and m/z = 18 (assigned to H_2O). Functional groups were identified by the decomposition temperature according to Figueiredo et al. [2].

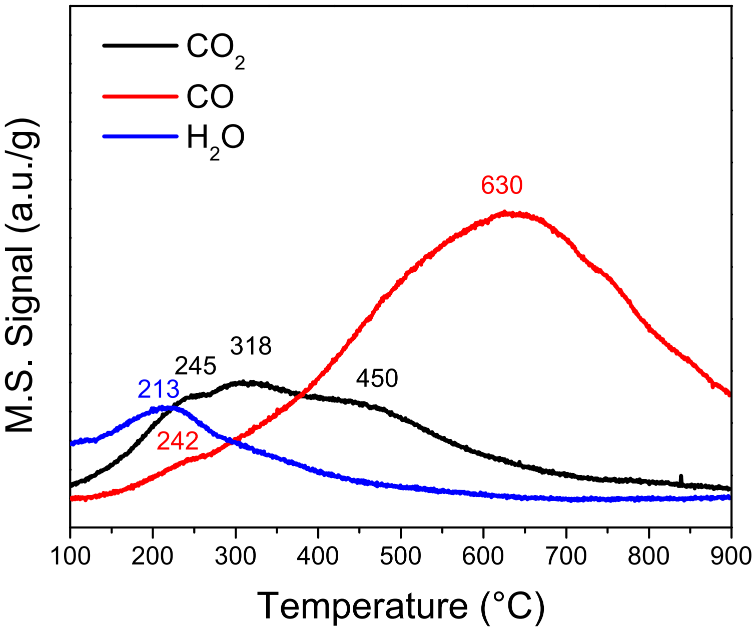

Figure S2. TPD-He of high surface graphite support

MS-signal (CH_4, CO_2 and CO) are shown in Figure S3 with the temperature-programmed reduction of rhenium-based catalysts supported on graphite. CH_4-MS signal is depicted in Fig 4b and is associated with the partial gasification of the graphite. Figure 4c and 4d shows between 200 °C-350 °C and 500 °C-900 °C a broad peak, respectively attributed to decomposition of OFG previously explained[1].

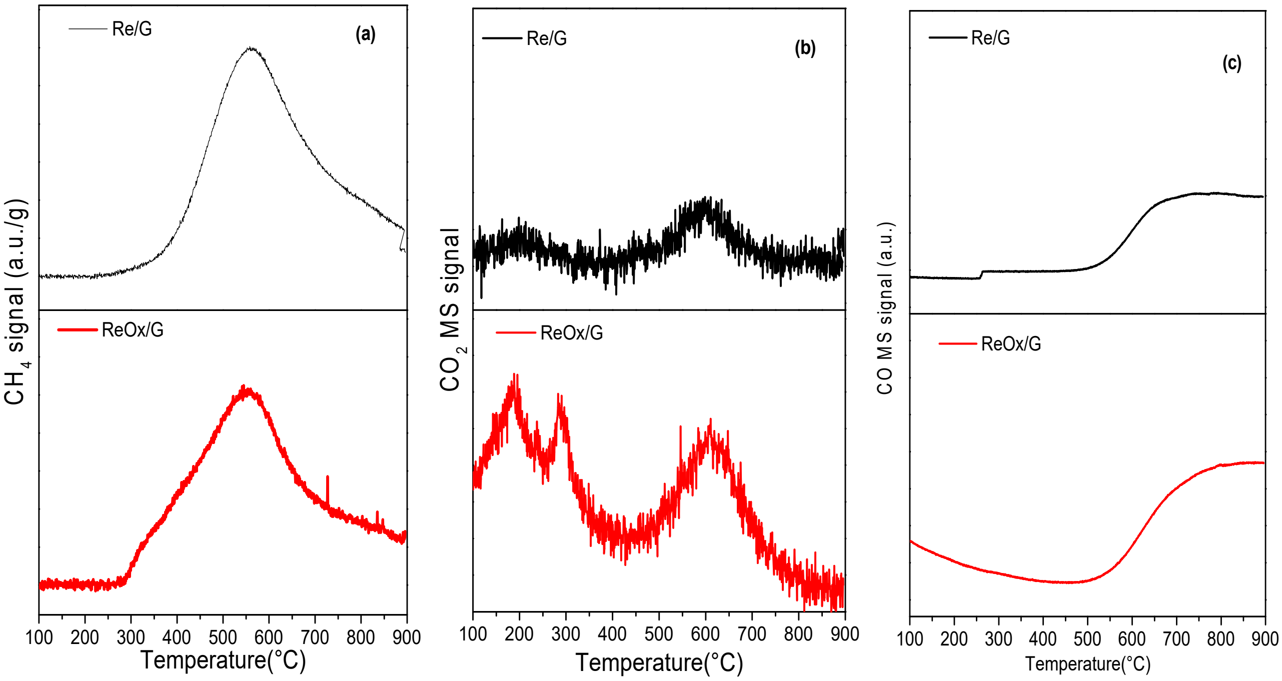

Figure S3: MS-signals of (a) CH_4, (b) CO_2, (c) CO observed during the TPR.

Table S1: Initial rate of hydrogenation of FAc

Samples	solvent	Temperature	**Pressure**	**r_0**($x10^{-6}$ mol·$gcat^{-1}$·s^{-1})	**Ref**
**Pd/MxNyO (M=Mg, Ca; N=Zr, Al)**	Acetone	120°C	55 bar	N.I.	[3]
Pd/C + HPW	Cyclohexane	130°C	10 bar	N.I.	[4]
Pt/TiO_2	Acetone	200°C	50 bar	N.I.	[5]
Pt/TiO_2	Acetone	200°C	50 bar	808	[6]
Re/G	H_2O	240°C	30 bar	17,3	This work

N.I.: Not Informed

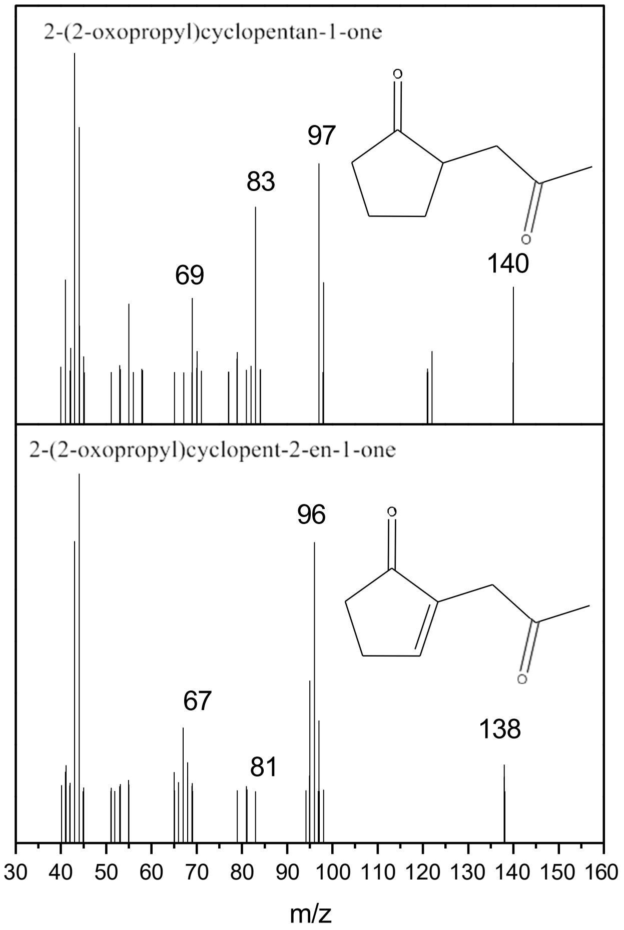

Figure S4: mass spectra of ring rearrangement products.

References

[1] E. Blanco *et al.*, "Effect of carbon support and functionalization on the synthesis of rhenium carbide and its use on HDO of guaiacol," *Catal Today*, vol. 420, p. 114031, Aug. 2023, doi: 10.1016/J.CATTOD.2023.02.008.

[2] J. L. Figueiredo, M. F. R. Pereira, M. M. A. Freitas, and J. J. M. Órfão, "Modification of the surface chemistry of activated carbons," *Carbon N Y*, vol. 37, no. 9, pp. 1379–1389, Jan. 1999, doi: 10.1016/S0008-6223(98)00333-9.

[3] L. Faba, E. Díaz, and S. Ordóñez, "Performance of bifunctional Pd/MxNyO (M=Mg, Ca; N=Zr, Al) catalysts for aldolization–hydrogenation of furfural–acetone mixtures," *Catal Today*, vol. 164, no. 1, pp. 451–456, 2011, doi: https://doi.org/10.1016/j.cattod.2010.11.032.

[4] S. Li *et al.*, "One-pot hydrodeoxygenation of bioderived furans into octane at low temperatures via an octanediol route," *Green Chemistry*, vol. 23, no. 13, pp. 4741–4752, 2021, doi: 10.1039/D1GC00916H.

[5] M. Goepel, R. Ramos, R. Gläser, and D. Kubička, "Novel polymer-silica composite-based bifunctional catalysts for hydrodeoxygenation of 4-(2-Furyl)-3-Buten-2-One as model substance for furfural-acetone aldol condensation products," *Applied Sciences (Switzerland)*, vol. 9, no. 12, Jun. 2019, doi: 10.3390/app9122438.

[6] R. Ramos, Z. Tišler, O. Kikhtyanin, and D. Kubička, "Solvent effects in hydrodeoxygenation of furfural-acetone aldol condensation products over Pt/TiO2 catalyst," *Appl Catal A Gen*, vol. 530, pp. 174–183, Jan. 2017, doi: 10.1016/J.APCATA.2016.11.023.
